# Supplementary material for: Evaluating inositol phospholipid interactions with inward rectifier potassium channels and characterising their role in disease
Source: Commun Chem. 2020 Oct 30;3:147. doi: 10.1038/s42004-020-00391-0 (PMC9814360; doi:10.1038/s42004-020-00391-0)
Supplement: Supplementary file 3 — Supplementary Information [file 42004_2020_391_MOESM3_ESM.pdf]

Evaluating Inositol phospholipid interactions with Inward Rectifier Potassium Channels and characterising their role in Disease

Tanadet Pipatpolkai[1,2,3], Robin A. Corey[2], Peter Proks[1,3], Frances M. Ashcroft[1,3]*, Phillip J. Stansfeld[2,3,4]*

[1]Department of Physiology Anatomy and Genetics, Parks Road, Oxford, OX1 3PT, UK.

[2]Department of Biochemistry, South Parks Road, Oxford, OX1 3QU, UK.

[3]OXION Initiative in Ion Channels and Disease, University of Oxford, Oxford OX1 3PT, UK.

[4]School of Life Sciences & Department of Chemistry, University of Warwick, Coventry, CV4 7AL, UK.

*corresponding authors

Keywords: Inwardly rectifying channel, molecular dynamics

Supplementary information

Supplementary figure 1

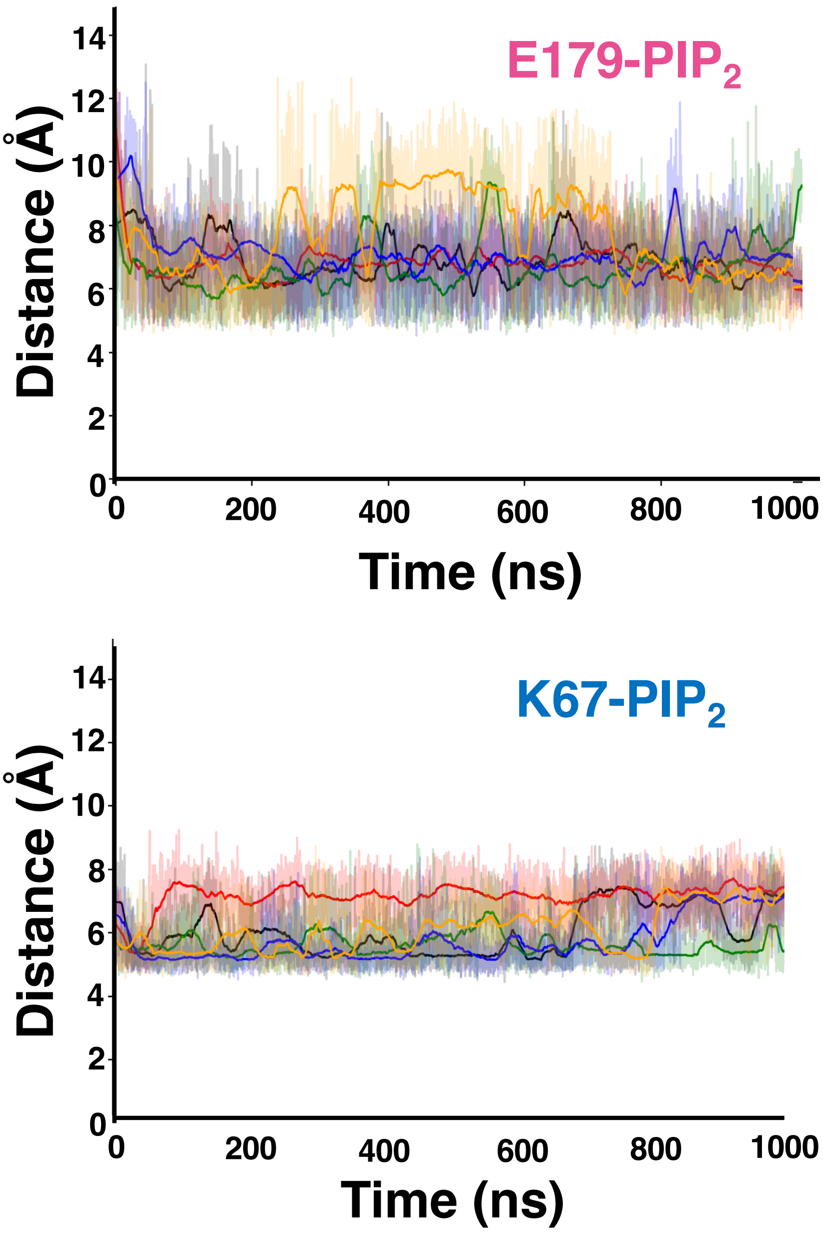

Supplementary Figure 1 Distance between protein backbone and the PIP_2 headgroup

Calculated distance between the PIP_2 headgroup and the backbone of either **a** E179 or **b** K67, over a 1 µs simulation. The different colours indicate the individual repeats of the simulation. The darker lines show the running average for each simulation (n=5).

Supplementary figure 2

PI -> PC

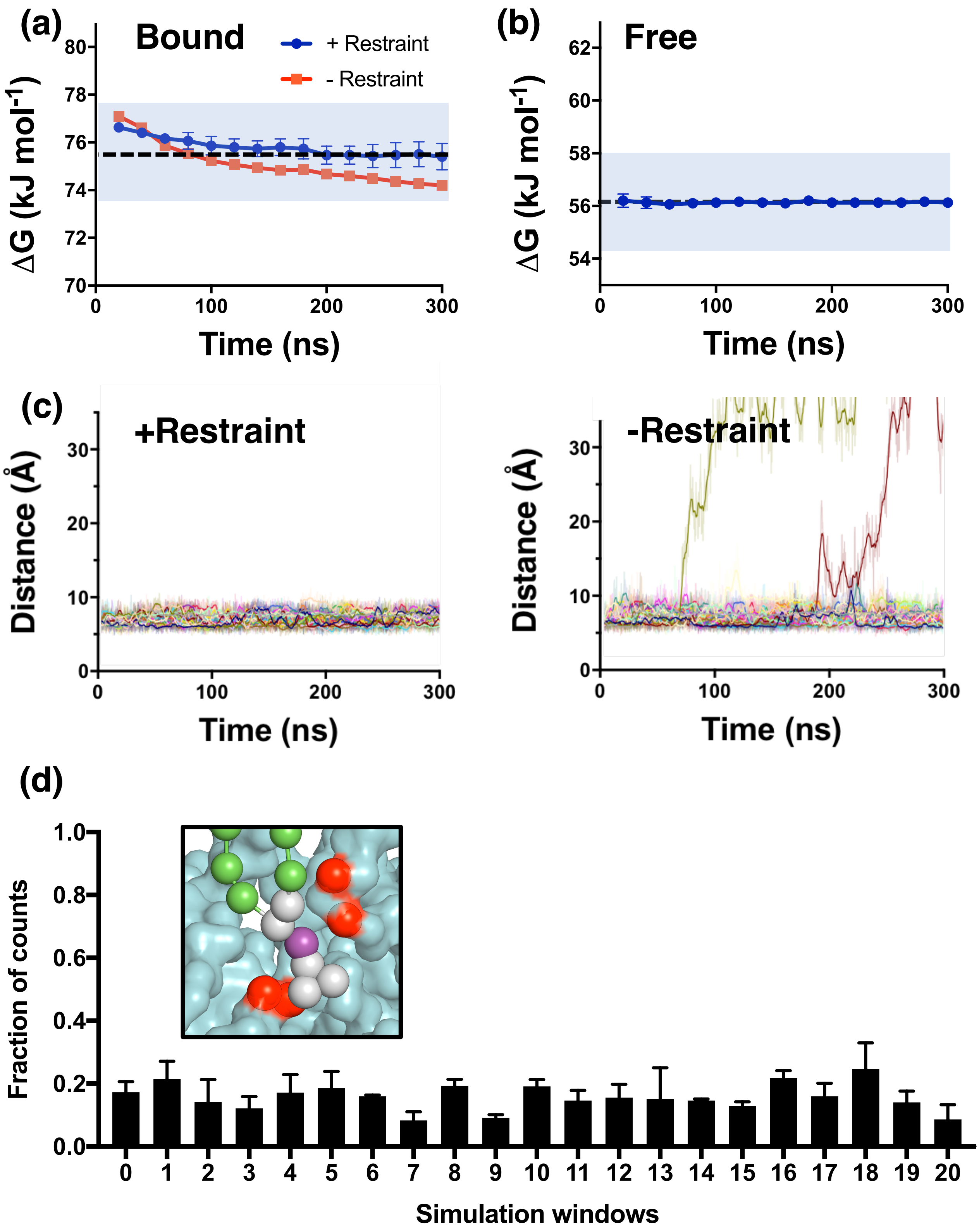

Supplementary Figure 2 Convergence analysis of PI to PC free energy perturbation.

a Convergence of the free energy calculation during PI to PC perturbation in the presence of hKir6.2. The analysis was carried out with (blue) and without (red) the flat-bottom restraint. The blue shaded region illustrates the thermal fluctuation of the system, i.e. kT.

b Convergence of the free energy calculation during PI to PC perturbation in the bulk PC bilayer in the absence of hKir6.2. The blue shaded region illustrates the thermal fluctuation of the system, i.e. kT.

c The distance between the phosphate headgroup (PO4 particle) and the average position of four amino acid residues (red), in the presence and absence of a flat-bottom restraint. These four residues were chosen as they are 6 Å away from the lipid headgroup. Different colours represent the simulations in the different alchemical states (λ windows) of PI to PC transformation.

d Fraction of counts where the PO4 particle experiences the flat-bottom restraint in each simulation window. Inset: A flat bottom restraint was imposed between the PO4 particle (purple) and the protein backbone (red).

Supplementary figure 3

PIP$_2$ -> PC

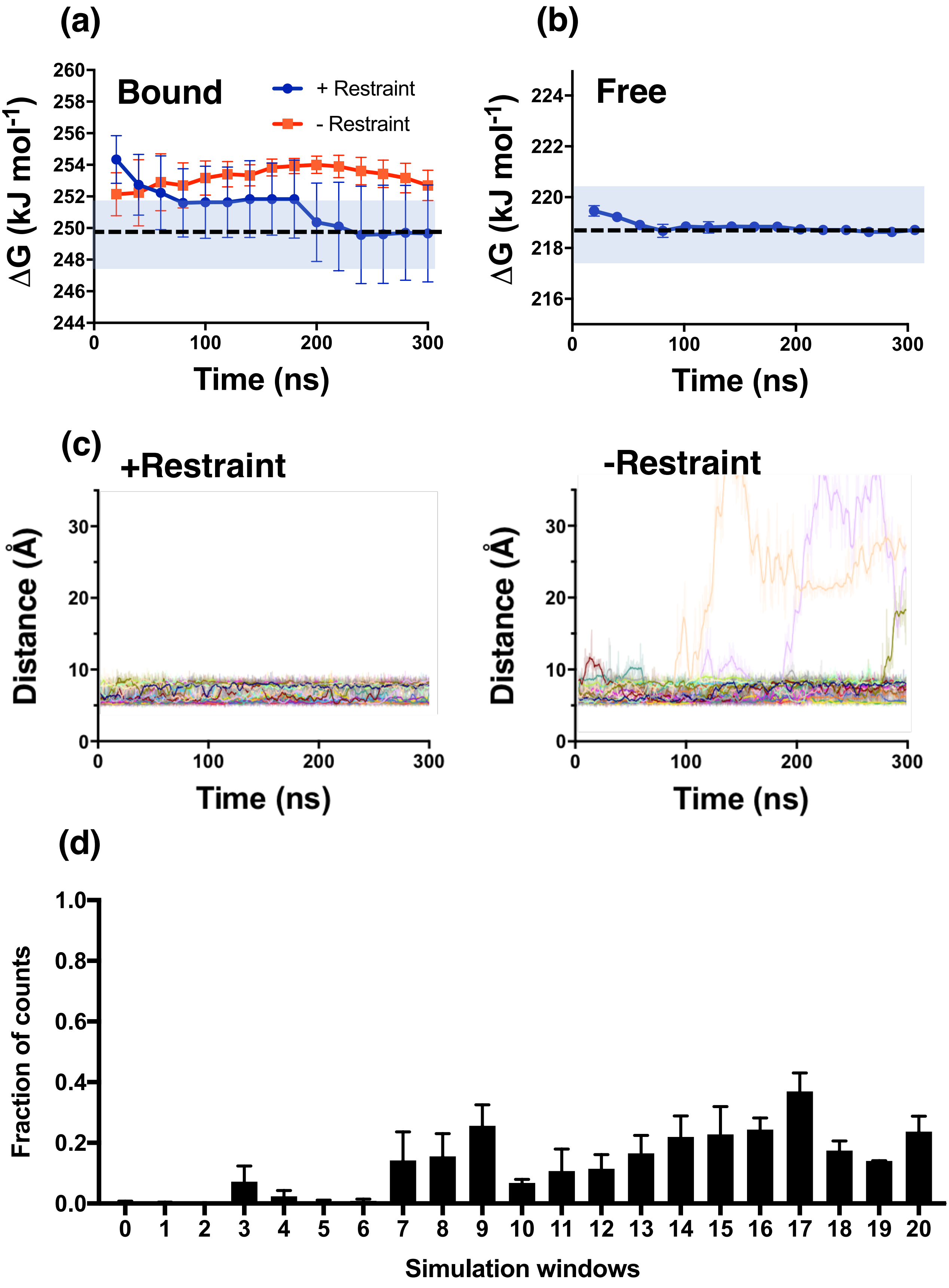

Supplementary Figure 3 Convergence analysis of PIP_2 to PC free energy perturbation.

a Convergence of the free energy calculation during PIP_2 to PC perturbation in the presence of hKir6.2. The analysis was carried out with (blue) and without (orange) the flat-bottom restraint. The blue shaded region illustrates the thermal fluctuation of the system, i.e. kT.

b Convergence of the free energy calculation during PIP_2 to PC perturbation in the bulk PC bilayer. The blue shaded region illustrates the thermal fluctuation of the system, i.e. kT.

c The distance between the phosphate headgroup (PO4 particle) and the centre position between the backbone of 4 amino acid residues (red) in the presence and absence of a flat-bottom restraint. These residues were chosen as they are 6 Å away from the lipid headgroup. Different colours represent the simulations in the different alchemical states (λ windows) of PIP_2 to PC transformation.

d Fraction of counts where the PO4 particle experiences the flat-bottom restraint in each simulation windows.

Supplementary figure 4

PIP_2 -> PI4P

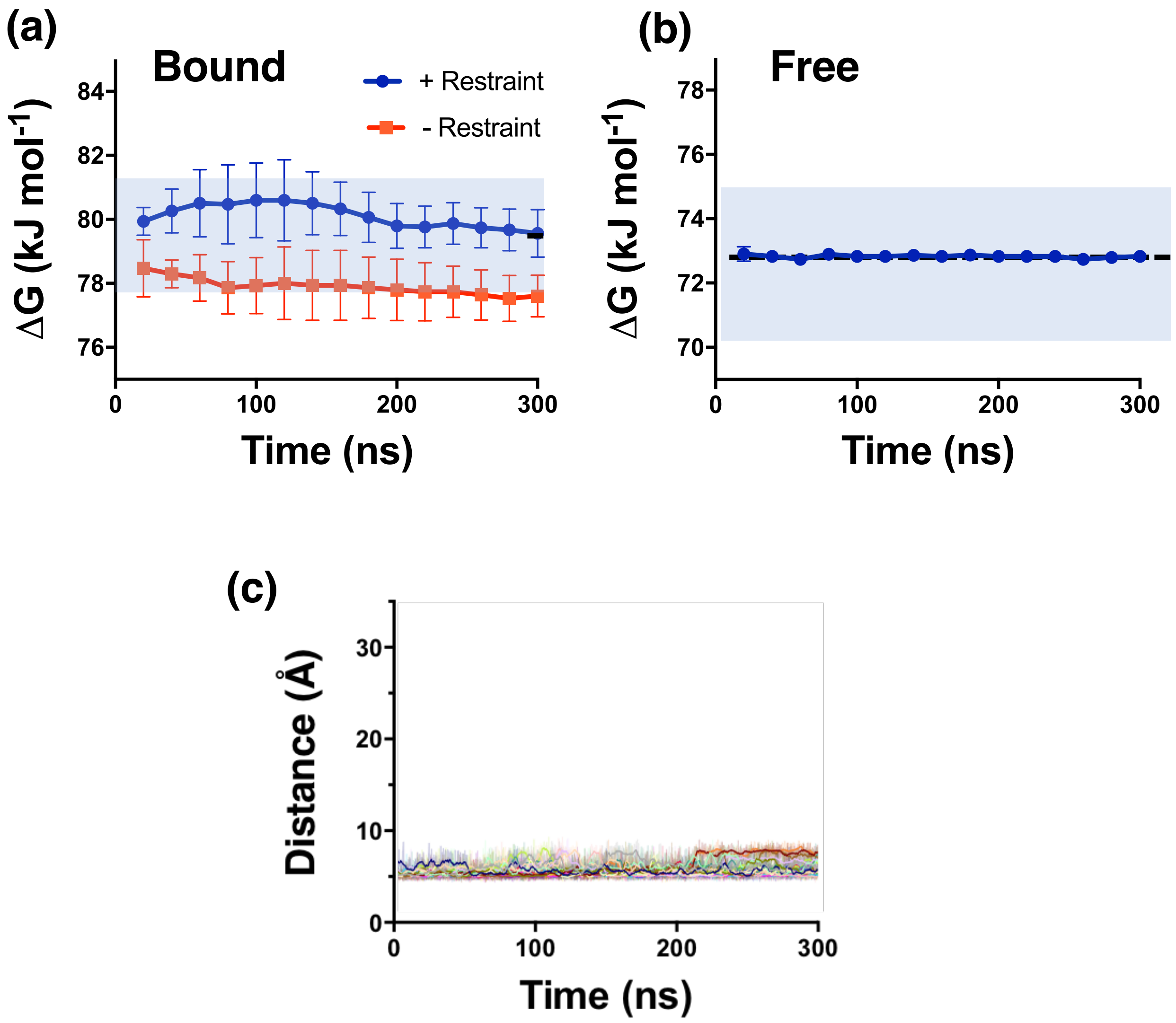

Supplementary Figure 4 Convergence analysis of PIP_2 to PI4P free energy perturbation.

a Convergence of the free energy calculation during PIP_2 to PI4P perturbation in the presence of hKir6.2. The analysis was carried out with (blue) and without (orange) the flat-bottom restraint. The blue shaded region illustrates the thermal fluctuation of the system, i.e. kT.

b Convergence of the free energy calculation during PIP_2 to PI4P perturbation in the bulk PC bilayer. The blue shaded region illustrates the thermal fluctuation of the system, i.e. kT.

c The distance between the phosphate headgroup (PO4 particle) and the flat-bottom restraint. Different colours represent the individual simulations in the different alchemical states (λ windows) of the PIP_2 to PI4P transformation.

Supplementary figure 5

PI4P -> PI

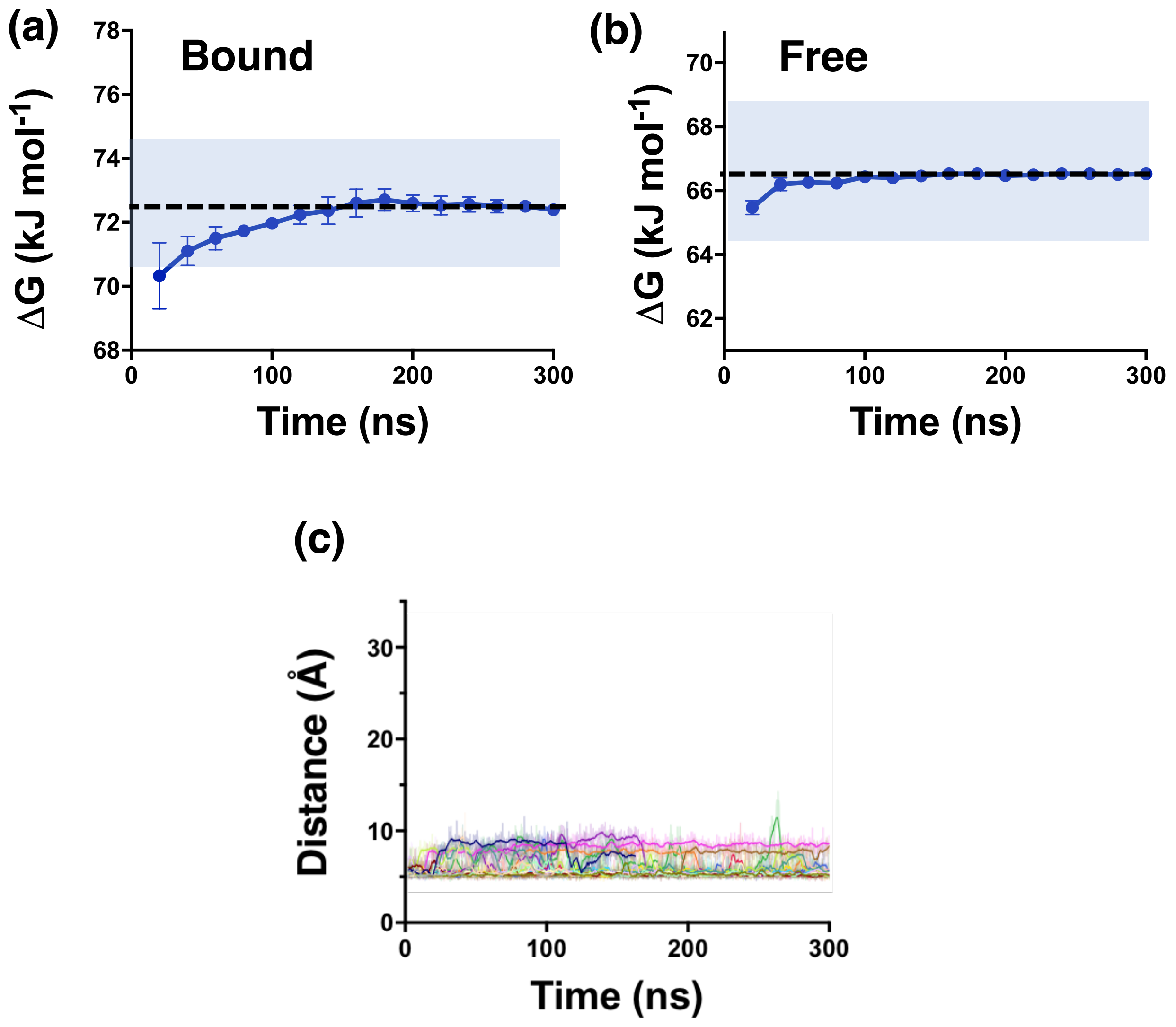

Supplementary Figure 5 Convergence analysis of PI4P to PI free energy perturbation.

a Convergence of the free energy calculation during PI4P to PI perturbation in the presence of hKir6.2. The analysis was carried out with (blue) and without (orange) the flat-bottom restraint. The blue shaded region illustrates the thermal fluctuation of the system, i.e. kT.

b Convergence of the free energy calculation during PI4P to PI perturbation in the bulk PC bilayer. The blue shaded region illustrates the thermal fluctuation of the system, i.e. kT.

c The distance between the phosphate headgroup (PO4 particle) and the flat-bottom restraint. Different colours represent the simulations in the distinct alchemical states (λ windows) of the PI4P to PI transformation.

Supplementary figure 6

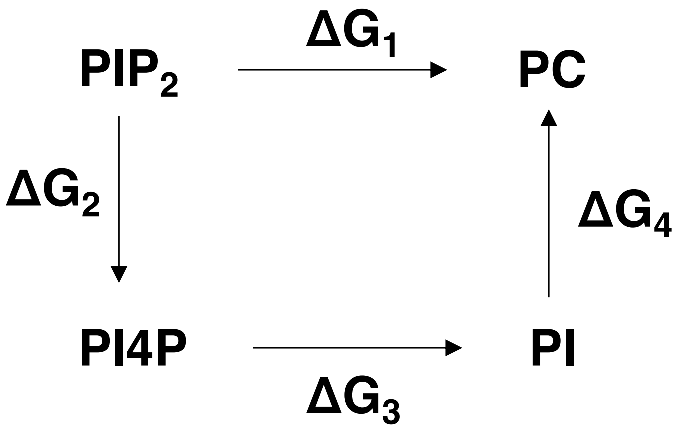

$$\Delta G_1 = \Delta G_2 + \Delta G_3 + \Delta G_4$$
$$\Delta G_1 = 5 + 6 + 19$$
$$\Delta G_1 = 30 \text{ kJ mol}^{-1}$$

$$\Delta G_{1(calc)} = 31 \text{ kJ mol}^{-1}$$

Supplementary Figure 6 A complete thermodynamic cycle in PIP_2 stepwise perturbation

The thermodynamic cycle representing relative binding free energy between phosphoinositide lipids (PIP_2, PI4P and PI) and PC.

Supplementary figure 7

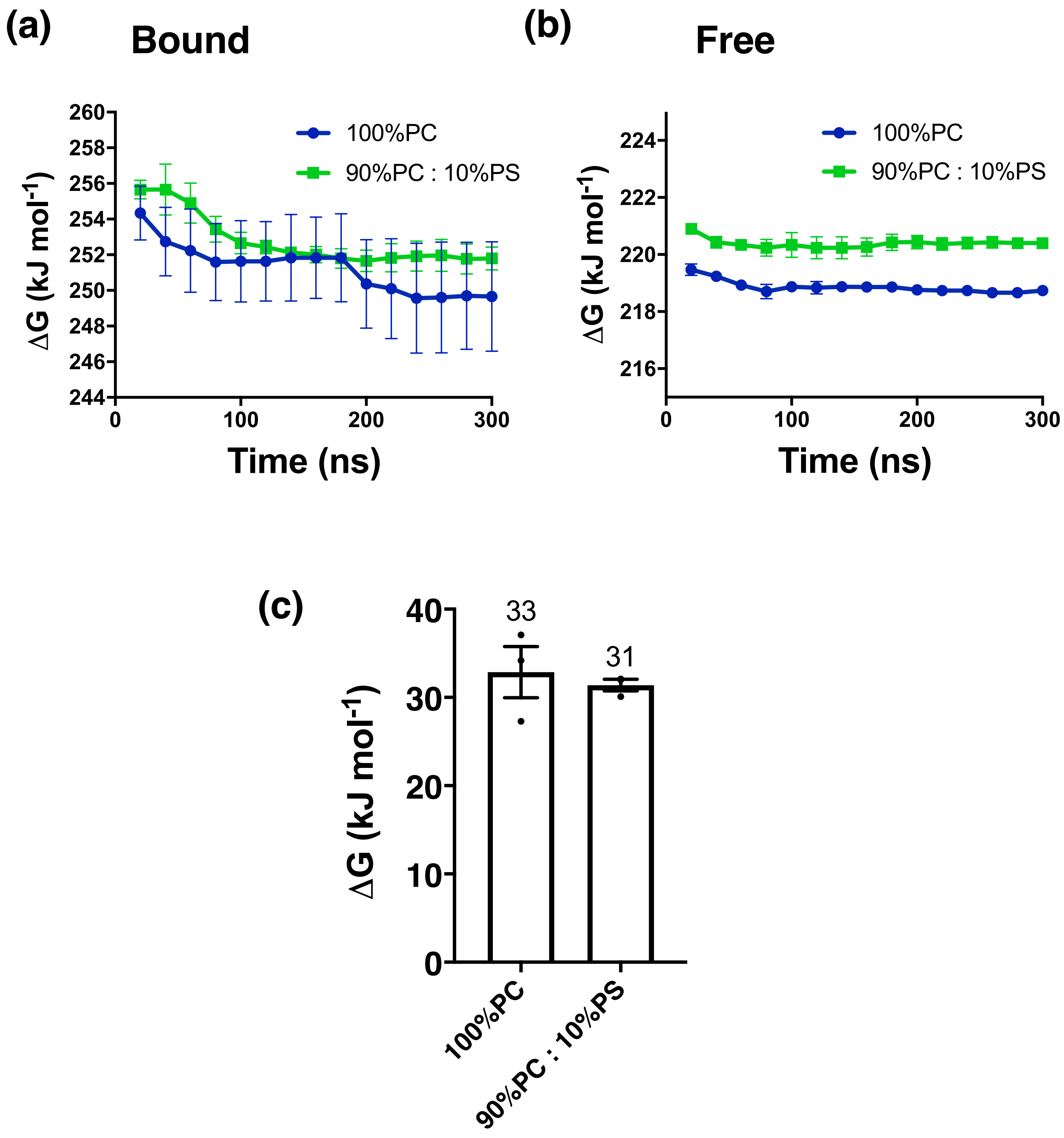

Supplementary Figure 7 Convergence analysis of PIP_2 to PC free energy perturbation in anionic lipid environment.

- **a** Convergence of the free energy calculation during PIP_2 to PC perturbation in the presence of hKir6.2. The analysis was carried out in 100% PC bilayer (blue) or mixed bilayer containing 10% PS (green)
- **b** Convergence of the free energy calculation during PIP_2 to PC perturbation in the bulk PC bilayer. The analysis was carried out in 100% PC bilayer (blue) or mixed bilayer containing 10% PS (green)
- **c** Binding free energy between PIP_2 and Kir6.2 in 100% PC bilayer or in 10% PS. Values are rounded to the nearest whole number. Error bars represent the SEM (n=3)

Supplementary figure 8

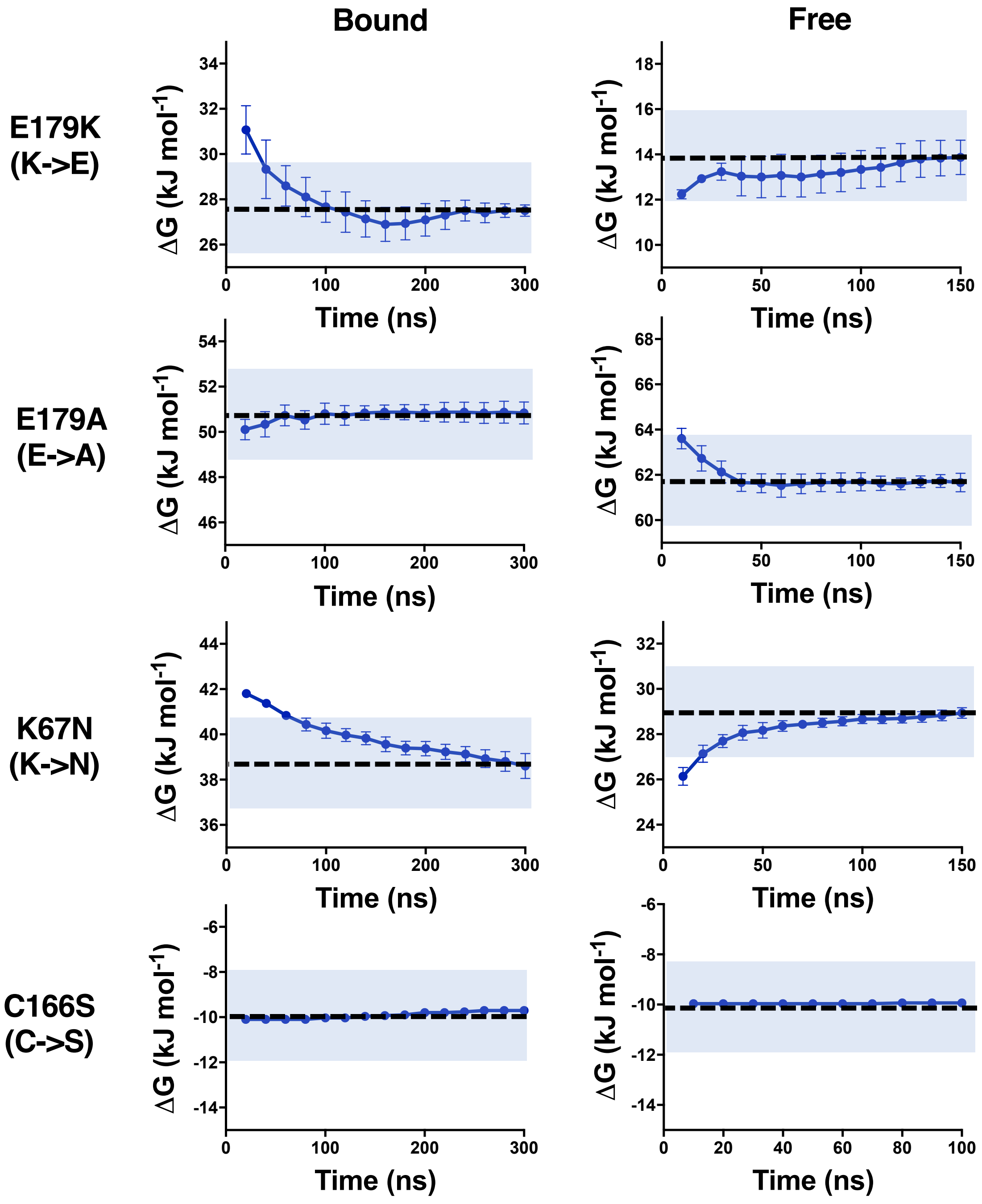

Supplementary Figure 8 Convergence analysis of E179K (K→E), E179A, K67N and C166S free energy perturbation.

Convergence of the free energy calculation during lysine to glutamate perturbation for residue 179 (E179K), glutamate to alanine perturbation for residue 179 (E179A), lysine to asparagine perturbation for residue 67 (K67N) and cysteine to serine perturbation for position 166 (C166S) of hKir6.2 in (a) the presence and (b) the absence of PIP_2. The shaded blue region illustrates the thermal fluctuation of the system, i.e. kT.

Supplementary figure 9

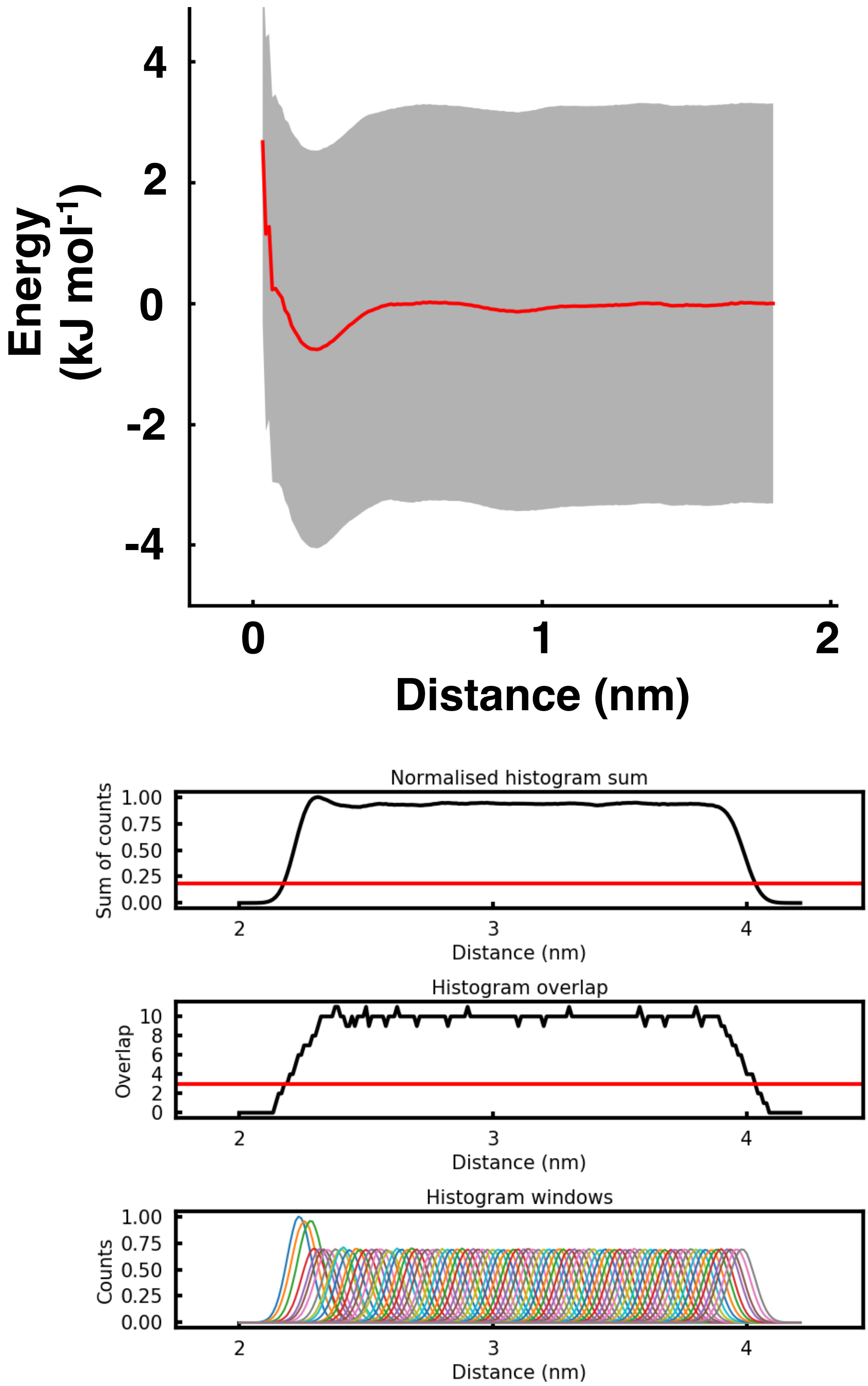

Supplementary Figure 9 PMF data for PC binding to hKir6.2

The 1D energy landscape for PIP_2 binding to hKir6.2. The y-axis is set to 0 in PC and the x-axis is set to 0 nm. The calculated ΔG is ca. 0 kJ/mol.

Supplementary figure 10

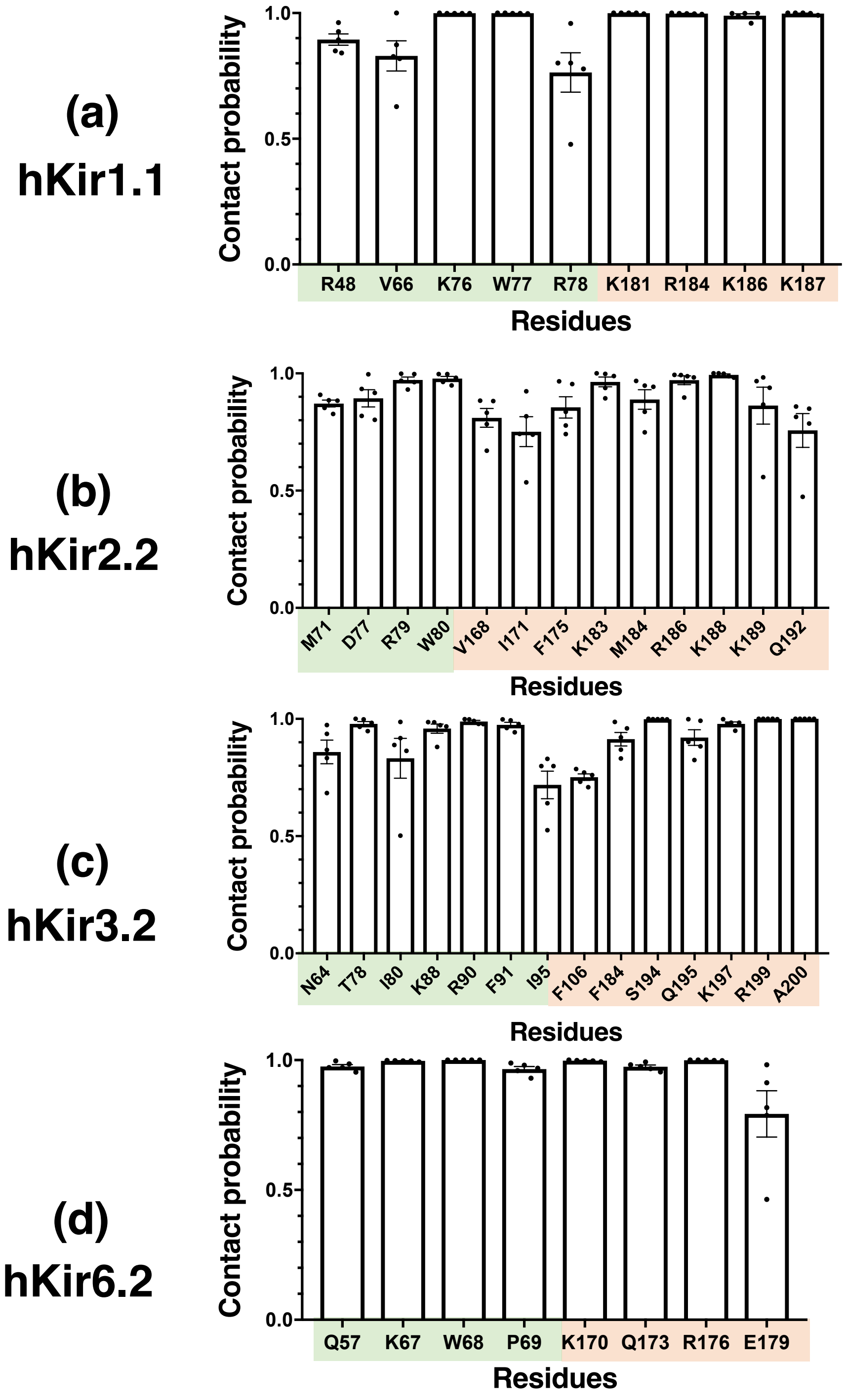

Supplementary Figure 10 A PIP_2 binding site on hKir channels

PIP_2 contact analysis showing residues which make contact >75% of the time during a 1 µs simulation for **a** hKir1.1, **b** hKir2.2, **c** hKir3.2 or **d** hKir6.2 channels (n = 5). Residues in the N-terminal domain are highlighted in green and in the C-terminal domain are coloured in orange.

Supplementary Tables

PDB ID	Resolution (Å)	Conformation	Ligand(s)	References
5WUA	5.6	Closed (Propeller)	none added	Li 2017
5TWV	6.3	Closed (Propeller)	ATP	Martin 2017a
6BAA	3.6	Closed (Propeller)	ATP and Glibenclamide	Martin 2017b
6C3O	3.9	Closed (Quatrefoil)	ATP and ADP	Lee 2017
6C3P	5.6	Closed (Propeller)	ATP and ADP	Lee 2017
5YW8	4.4	Closed (Propeller)	ATPγS	Wu 2018
6JB1	3.3	Closed (Propeller)	Repaglinide and ATPγS	Ding 2019
6PZ9	3.7	Closed (Propeller)	Repaglinide and ATP	Martin 2019

Supplementary Table 1 List of all cryo-EM K_{ATP} channel structures

Table of K_{ATP} channel structures giving their PDB ID, overall resolution, conformation and bound ligands.

Transformation		ΔΔG (kJ/mol)
From	To	
PIP_2	PI4P	5 ± 1
PI4P	PI	6 ± 0
PI	PC	19 ± 0
PIP_2	PC	33 ± 3
PIP_2	PIP_2-diC8	-2 ± 0

Supplementary Table 2 Calculation of the ΔΔG on an individual phosphate group and the fatty acid chains on a hKir6.2 tetramer

Change in binding free energy (ΔΔG) when individual phosphate groups are perturbed (i.e. from PIP_2 to PI4P, from PI4P to PI and from PI to PC (values in black), when PIP_2 is perturbed to PC and when PIP_2 is perturbed to PIP_2-diC8. Values are rounded to the nearest whole number (n=3).

Mutation	-ΔΔG (kJ mol^{-1})
E179K (K→E)	14 ± 1
E179A	11 ± 1
K67N	-10± 1
C166S	0 ± 0

Supplementary Table 3 Free energy calculations using disease associated hKir6.2 mutations.

The energetic cost of making the residue mutation based on the schematic diagram (Figure 3b).

**Protein**	**ΔΔG (kJ mol^{-1})**
hKir6.2	33 ± 3
hKir6.2 + SUR1	38 ± 2

Supplementary Table 4 Free energy calculations using PIP_2 to PC transformation

Binding free energy changes between wild-type and mutated hKir6.2 channels and hKir6.2 channel with SUR1 as we perturbed PIP_2 to PC. Values are rounded to the nearest whole number. Mean and SEM (n=3).

Protein	ΔΔG (kJ/mol)
hKir1.1	53 ± 1
hKir2.2	41 ± 1
hKir3.2	47 ± 1
hKir6.2	33 ± 3

Supplementary Table 5 Free energy calculation on different hKir channels

Binding free energy changes between different hKir channels as we perturbed PIP_2 to PC. Values are rounded to the nearest whole number. Mean and SEM (n=3).

Protein	Transformation		ΔΔG (kJ/mol)
	From	To	
hKir1.1	PIP_2	PI4P	13 ± 1
	PI4P	PI	16 ± 0
	PI	PC	23 ± 0
	**Sum**		**52 ± 1**
hKir2.2	PIP_2	PI4P	6 ± 0
	PI4P	PI	13 ± 0
	PI	PC	21 ± 1
	**Sum**		**40 ± 1**
hKir3.2	PIP_2	PI4P	7 ± 0
	PI4P	PI	11 ± 1
	PI	PC	24 ± 0
	**Sum**		**42 ± 1**
hKir6.2	PIP_2	PI4P	5 ± 1
	PI4P	PI	6 ± 0
	PI	PC	19 ± 0
	**Sum**		**30 ± 1**

Supplementary Table 6 Calculation of the ΔΔG on an individual phosphate group on hKir channels.

Change in binding free energy (ΔΔG) when individual phosphate groups are perturbed (i.e. from PIP_2 to PI4P, from PI4P to PI and from PI to PC (values in black), The sum of these values are shown in bold. Values are rounded to the nearest whole number (n=3).

Protein	PIP_2	POPC	Water	Na	Cl	Box size
hKir1.1	1	495	14196	314	330	134x134x149 $Å^3$
hKir2.2	1	538	16324	378	353	140x140x155 $Å^3$
hKir3.2	1	463	12451	321	285	131x131x141 $Å^3$
hKir6.2	1	508	13989	328	308	135x137x145 $Å^3$

Supplementary Table 7 Number of molecules in the simulation system.

The number of PIP_2, POPC, Water, Na and Cl and the box size used in the unbiased simulation and the free energy calculation.

Mutation	Direction of Perturbation	From		To	
		SC1	SC2	SC1	SC2
K67N	WT → K67N	C3	Qd	P5	Dum
C166S	WT → C166S	C5	-	P1	-
E179K	E179K → WT	C3	Qd	Qa	Dum
E179A	WT → E179A	Qa	-	Dum	-

Supplementary Table 8 Direction of free energy perturbation of amino acid residues

The direction of the transformation and the particle types for free energy perturbation analysis. The particles are suggested based on MARTINI2.2 forcefield. Dum is a dummy particle with a mass of 72 and no bonded or non-bonded interactions.
